# Radiographic progression-free survival as a surrogate endpoint for overall survival in first-line ARPi naïve metastatic castration-resistant prostate cancer

**DOI:** 10.1093/oncolo/oyaf425

**Published:** 2026-01-06

**Authors:** Elena Castro, Stefanie Paganelli, Di Wang, Anja Haltner, Alexander Niyazov, Jane Chang, Imtiaz A Samjoo, Pedro C Barata

**Affiliations:** Department of Medical Oncology, Hospital Universitario 12 de Octubre, Madrid 28041, Spain; Value & Evidence, EVERSANA™, Burlington, ON, L7N 3H8, Canada; Value & Evidence, EVERSANA™, Burlington, ON, L7N 3H8, Canada; Data Analytics and Evidence Synthesis, EVERSANA™, New York, NY 10018, United States; Value & Evidence, Pfizer, Inc., New York, NY 10001, United States; Value & Evidence, Pfizer, Inc., New York, NY 10001, United States; Value & Evidence, EVERSANA™, Burlington, ON, L7N 3H8, Canada; University Hospitals Seidman Cancer Center, Case Comprehensive Cancer Center, Cleveland, OH 44106, United States

**Keywords:** metastatic castration-resistant prostate cancer, radiographic progression-free survival, overall survival, correlation analysis, systematic literature review

## Abstract

**Background:**

Overall survival (OS) is the gold standard endpoint in oncology trials but requires long follow-up and may be confounded by post-protocol treatments. Radiographic progression-free survival (rPFS) is used as an earlier endpoint in metastatic castration-resistant prostate cancer (mCRPC). This study evaluated the validity of rPFS as a surrogate for OS in first-line, asymptomatic/mildly symptomatic, androgen receptor pathway inhibitor (ARPi) naïve, mCRPC using methods recommended by Germany’s Institute for Quality and Efficiency in Health Care (IQWiG).

**Materials and Methods:**

A systematic search in Ovid^®^ identified randomized controlled trials reporting both rPFS and OS. Trial-level rPFS-OS correlations of hazard ratios (HRs) were calculated using bivariate random-effects meta-analysis (BRMA) and weighted linear regression (WLR). Correlation strength was interpreted per IQWiG criteria. The surrogate threshold effect (STE) was estimated to assess surrogacy. Leave-one-out cross-validation (LOOCV) assessed model robustness. The primary analysis included trials meeting the proportional hazards (PH) assumption. Sensitivity analyses included trials violating PH and further excluded outliers outside the 95% confidence intervals (CIs) in the correlation plot.

**Results:**

Eleven RCTs were identified. The primary analysis (*n* = 10 trials) yielded medium correlations (BRMA R^2^: 0.78 [95% CI: 0.53-0.90]; WLR R^2^: 0.65 [0.40-0.90]; STE: 0.83). Sensitivity analyses yielded medium (*n* = 11 trials) and strong (*n* = 8 trials) correlations. LOOCV showed good predictive accuracy (75%-82%).

**Conclusion:**

Results suggest rPFS is a valid surrogate for OS in first-line ARPi naïve mCRPC per IQWiG criteria. A statistically significant OS effect can be inferred for a trial demonstrating an upper confidence limit of HR < 0.83 in rPFS.

Implications for Practice Due to the trade-offs in assessing overall survival (OS) from randomized trials, establishing intermediate clinical endpoints that can predict therapeutic benefit earlier may help accelerate approval of emerging therapies. Radiographic progression-free survival (rPFS) has been increasingly used as a meaningful imaging-based primary endpoint in clinical trials for metastatic castration-resistant prostate cancer (mCRPC). Using the methodology recommended by the German health authority IQWiG, this study supports rPFS as a valid intermediate endpoint for OS in first-line ARPi naïve mCRPC.

## Introduction

Prostate cancer is the most common malignancy in men and the second leading cause of cancer-related death, accounting for 29% of new cancer diagnoses in men.^1^^,^^2^ First-line treatment for metastatic castration-resistant prostate cancer (mCRPC) includes a range of therapies with diverse mechanisms of action, such as taxane-based chemotherapy (eg, docetaxel), androgen receptor pathway inhibitors (ARPi) (eg, abiraterone acetate and enzalutamide), immunotherapy (eg, sipuleucel-T), α-emitters (eg, radium-223), and more recently, poly adenosine diphosphate-ribose polymerase inhibitors (PARPi), either alone or in combination with ARPis.

The approval of novel cancer treatments typically hinges on demonstrating improvements in overall survival (OS) through randomized controlled trials (RCTs). However, OS as a gold-standard endpoint presents challenges: it requires long follow-up periods, leading to high clinical trial costs, and may be confounded by post-progression treatments, including cross-over to the investigational agent. Additionally, waiting for OS data may result in missed opportunities for patients who could benefit from earlier approval of effective therapies.^3^ Conversely, approving treatments without long-term OS data may result in the use of therapies with limited clinical benefit, potentially putting patients at risk due to unknown long-term efficacy and safety profiles.^4^

To address these challenges, intermediate or surrogate endpoints can be explored to accelerate drug development. Assessing the correlational relationship between intermediate endpoints and OS can help predict long-term clinical outcomes earlier, accelerating decision-making and the incorporation of effective drugs into clinical practice. The Prostate Cancer Working Group (PCWG) has standardized rPFS criteria, and several trials have shown that rPFS correlates well with OS at the individual patient level.^5-8^ Based on these findings, regulatory agencies such as the United States Food and Drug Administration (FDA) have endorsed PCWG2-based rPFS, which has since been incorporated into most clinical trial designs.^9^ However, these correlations are primarily based on trials involving androgen-receptor inhibitors, and it remains unclear whether rPFS is a valid surrogate for OS across other therapeutic classes, including PARPi combinations.

Surrogate endpoints have become increasingly influential in health technology submissions, with regulatory authorities such as the European Medicines Agency (EMA) and the United States FDA granting approvals for drugs and biologics based on these indicators.^10^^,^^11^ Health Technology Assessment (HTA) agencies and regulatory bodies worldwide have issued guidance on evaluating the validity of surrogate endpoints.^12^^,^^13^ In Germany, the Institute for Quality and Efficiency in Health Care (IQWiG) recommends correlation-based methods with defined cut-off values for acceptable associations, and the European network for Health Technology Assessment (EUnetHTA) aligns with this guidance.^14^ In the United Kingdom, the National Institute for Health and Care Excellence (NICE) suggests that predictive accuracy is more important than correlation strength and recommends leave-one-out cross-validation (LOOCV) to assess surrogate validity.^10^ In contrast, the United States FDA does not prescribe statistical validation methods but provides a list of accepted surrogate endpoints and corresponding disease areas used in past approvals.^15^

Despite the widespread use of rPFS in mCRPC trials, its validity as a surrogate for OS in the first-line setting, particularly in asymptomatic or mildly symptomatic patients and in trials including PARPi combinations, has not been comprehensively evaluated at the trial level. The aim of this study was to evaluate the validity of rPFS as a surrogate endpoint for OS in first-line asymptomatic or mildly symptomatic mCRPC, using methodology recommended by IQWiG. To this end, we conducted a systematic literature search and trial-based correlation analysis from multiple RCTs in this setting.

## Methods

### Systematic literature review

#### Protocol and registration

A systematic literature review (SLR) of RCTs evaluating first-line treatments for patients with asymptomatic or mildly symptomatic mCRPC was conducted in accordance with the Preferred Reporting Items for Systematic Literature Reviews and Meta-Analyses (PRISMA) statement.^16^^,^^17^ The study protocol was developed in accordance with the PRISMA for systematic review protocols (PRISMA-P) statement and prospectively registered with the International Prospective Register of Systematic Reviews (PROSPERO; CRD42021283512).^18^^,^^19^ Additional SLR methods are provided in **Appendix A, including Tables S1 and S2, and Figure S1**. For the purposes of this research, RCTs identified through the search were further screened and included only if they met the predefined Population, Intervention, Comparator, Outcome, and Study Design (PICOS) criteria and reported relative treatment effects for both rPFS and OS, either as hazard ratios (HRs) or estimable from Kaplan-Meier curves.

### Statistical analysis

#### Outcomes

Endpoints of interest included rPFS and OS. For rPFS, the latest data cutoff available at the time of this analysis was used regardless of assessment by blinded independent central review (BICR) or investigator. When both investigator-assessed and BICR-assessed rPFS were available at the latest data cutoff, the BICR value was used.

#### Correlation analysis and assessment of surrogate endpoint

Trial-level correlation analyses based on HRs were conducted to evaluate the relationship between rPFS and OS. Hazard ratios were log transformed to be consistent with the linearity assumption for the relationship between treatment effects. The correlation between rPFS and OS was assessed using two ­methods: (1) bivariate random-effects meta-analysis (BRMA) model; ^10^^,^^20^ and (2) weighted linear regression (WLR) where studies were weighted based on their inverse variance.^21^ The degree of correlation between log HRs of rPFS and OS was assessed using the squared Pearson correlation coefficient (R^2^). The strength of the correlation estimates obtained from BRMA and WLR were assessed according to methods for validating surrogate endpoints as outlined in the IQWiG rapid report.^14^ According to IQWiG criteria, the strength of the correlation can be interpreted as follows: a high correlation is indicated by a lower limit of the 95% confidence interval (CI) with R ≥ 0.85 (or R^2^ ≥ 0.72); a medium correlation is indicated by R values between 0.7 and 0.85 (or R^2^ values between 0.49 and 0.72); and a low correlation is indicated by an upper limit of the 95% CI with R ≤ 0.7 (or R^2^ ≤ 0.49).^14^ To demonstrate the validity of a surrogate, a high correlation is required.^14^ In cases where no high correlation is evident, the validity of the surrogate remains unclear, and conclusions about patient-relevant endpoints can still be made by applying the surrogate threshold effect (STE) concept according to Burzykowski and Buyse.^14^^,^^22^ If the correlation is low, no statement regarding surrogate validation can be made.^14^ The STE is the minimum absolute value of the effect on the surrogate that must be observed in a new trial to deduce an effect on the clinical endpoint.^14^^,^^22^ In the current context, STE represents the maximum value of the HR for PFS (HR_rPFS_) needed to predict a significant effect on OS. In the case where medium correlations were observed, STEs were calculated to allow for conclusions to be made on surrogacy.

The validity and predictive accuracy of the estimated correlation equations from the BRMA model was assessed via LOOCV.^10^ In LOOCV, the model was formed by leaving out the treatment effect (log HR of OS) of one study. The OS value was then predicted using the study’s log HR of rPFS and the information from the remaining studies. The predicted OS was then compared to the reported OS to determine the model’s accuracy. This process was repeated for every study and an overall measure of accuracy was calculated by taking the proportion of reported HRs that fell within their respective 95% prediction interval. All statistical analyses were performed using R software (version 4.3.2).

#### Primary and sensitivity analyses

The primary analysis included all identified trials that met the proportional hazards (PH) assumption for both rPFS and OS. The first sensitivity analysis reintroduced trials in which the PH assumption was violated for either endpoint. A second sensitivity analysis was also conducted, which further excluded outlier trials. Outliers were defined as those falling outside the 95% CI in the scatterplot of log hazard ratios for rPFS and OS.

## Results

### Search results

In total, 7104 records were identified (after duplicates were removed) across all databases searched spanning from the original search date to the last search date. Of these, 6631 records were excluded at the title and abstract stage primarily because of population, interventions, or study types not of interest. After screening 455 full-text articles for eligibility, a total of 11 of these jointly reported rPFS and OS and were ultimately included in the current study (see **Tables S3 and S4, and Figures S2 to S5**).

### Characteristics of included studies

Study design and population characteristics of the included trials are summarized in Table 1. All 11 included studies were phase 3, multi-center RCTs, with nine being double-blind^23-31^ and two open-label.^32^^,^^33^ These studies assessed various first-line treatments for patients with asymptomatic or mildly symptomatic mCRPC.

**Table 1.** Summary of study characteristics for the included trials.

Trial	Phase	Setting	Blinding	Region	Treatments	Sample Size
**ACIS; NCT02257736^26^**	3	Multicenter	Double-blind	Multinational	Apalutamide 240 mg	492
					Abiraterone acetate 1000 mg	
					Prednisone 10 mg	
					Placebo	490
					Abiraterone acetate 1000 mg	
					Prednisone 10 mg	
**Alliance A031201; NCT01949337^33^**	3	Multicenter	Open-label	Multinational	Enzalutamide 160 mg	657
					Enzalutamide 160 mg	654
					Abiraterone acetate 1000 mg	
					Prednisone 5 mg	
**COU-AA-302; NCT00887198^23^ ^,^ ^34^**	3	Multicenter	Double-blind	Multinational	Abiraterone acetate 1000 mg	546
					Prednisone 5 mg	
					Placebo	542
					Prednisone 5 mg	
**PEACEIII; NCT02194842^32^**	3	Multicenter	Open-label	Multinational	Radium-223 55 kBq/kg	222
					Enzalutamide 160 mg	
					Enzalutamide 160 mg	224
**ERA 223; NCT02043678^27^**	3	Multicenter	Double-blind	Multinational	Radium-223 55 kBq/kg	401
					Abiraterone acetate 1000 mg	
					Prednisone or prednisolone 5 mg	
					Placebo	405
					Abiraterone acetate 1000 mg	
					Prednisone or prednisolone 5 mg	
**IPATential150; NCT03072238^28^**	3	Multicenter	Double-blind	Multinational	Ipatasertib 400 mg	547
					Abiraterone acetate 1000 mg	
					Prednisone 5 mg	
					Placebo	554
					Abiraterone acetate 1000 mg	
					Prednisone 5 mg	
**KEYNOTE-641; NCT03834493^31^**	3	Multicenter	Double-blind	Multinational	Pembrolizumab 200 mg IV Q3W	245^a^
					Enzalutamide 160 mg	
					Placebo IV Q3W	242^a^
					Enzalutamide 160 mg	
**NCT02294461^24^**	3	Multicenter	Double-blind	Asia	Enzalutamide 160 mg	198
					Placebo	190
**PREVAIL; NCT01212991^25^ ^,^ ^48^**	3	Multicenter	Double-blind	Multinational	Enzalutamide 160 mg	872
					Placebo	845
**PROpel; NCT03732820^29^**	3	Multicenter	Double-blind	Multinational	Olaparib 300 mg	399
					Abiraterone acetate 1000 mg	
					Prednisone or prednisolone 5 mg	
					Abiraterone acetate 1000 mg	397
					Prednisone or prednisolone 5 mg	
**TALAPRO-2; NCT03395197^30^ ^,^ ^49^**	3	Multicenter	Double-blind	Multinational	Talazoparib 0.5 mg	402
					Enzalutamide 160 mg	
					Placebo	403
					Enzalutamide 160 mg	

Abbreviations: mCRPC = metastatic castration-resistant prostate cancer; mg = milligram; kBq = kilobecquerel; kg = kilogram.

a The subgroup of patients who had not received prior abiraterone acetate was selected to align with the first-line mCRPC population of interest.

Five trials investigated ARPis. The COU-AA-302 trial investigated abiraterone acetate versus placebo.^23^ Both the PREVAIL and NCT02294461 trials investigated enzalutamide versus placebo.^24^^,^^25^ The ACIS trial investigated apalutamide in combination with abiraterone acetate versus placebo and abiraterone acetate.^26^ The Alliance A031201 trial investigated enzalutamide versus enzalutamide in combination with abiraterone acetate.^33^ Two trials investigated radiotherapy radium-223 in combination with ARPi versus ARPi. The ERA 223 trial investigated radium-223 in combination with abiraterone acetate versus placebo plus abiraterone acetate.^27^ The PEACEIII trial investigated radium-223 in combination with enzalutamide versus enzalutamide alone.^32^ The IPATential150 trial investigated the protein kinase B (AKT) inhibitor ipatasertib in combination with abiraterone acetate versus placebo and abiraterone acetate.^28^ The KEYNOTE-641 trial investigated immunotherapy pembrolizumab in combination with enzalutamide versus placebo and enzalutamide.^31^ Lastly, two trials, TALAPRO-2 and PROpel, investigated PARPi (talazoparib and olaparib) in combination with ARPi (enzalutamide and abiraterone acetate) versus placebo plus ARPi.^29^^,^^30^

Eligibility criteria were generally consistent across the 11 RCTs. All patients were required to have histologically or cytologically confirmed mCRPC, asymptomatic or mildly symptomatic disease and progressive disease at study entry with Eastern Cooperative Oncology Group (ECOG)  ≤ 1. Patients were also required to be adults (≥18 years), surgically or medically castrated and treatment-naive in the mCRPC state. Of note, no symptomatic criteria were required for enrollment in the PROpel trial; as such, a proportion of patients in each arm were symptomatic (defined as those with a Brief Pain Inventory—Short Form [BPI-SF] score ≥ 4 and/or opiate use). Additionally, the KEYNOTE-641 trial included patients previously treated with abiraterone in the metastatic hormone-sensitive prostate cancer (mHSPC) or first-line mCRPC state. Data were reported for the overall population and for those who had never received abiraterone. However, data for patients who received abiraterone only in the HSPC state were not reported. Therefore, data from the subgroup of patients with no prior abiraterone treatment were used to best match the populations of the other included trials. Life expectancy requirements varied across trials which ranged from ≥6  to ≥12 months and two trials did not report criterion for life expectancy.^24^^,^^26^

Among the 11 studies, five reported rPFS as the primary outcome,^26^^,^^28-30^^,^^32^ one reported OS as the primary outcome,^33^ and three reported OS as the co-primary outcome together with rPFS.^25^^,^^31^^,^^34^ In cases where rPFS or OS were not the primary outcomes, it was reported as time to PSA progression (NCT02294461)^24^ and symptomatic skeletal event free survival (ERA 223).^27^ The definition of OS was consistent across the included trials, where OS was broadly defined as the time from randomization to death from any cause. The definition of rPFS was consistent across the included trials, where rPFS was broadly defined as the time from randomization to first objective evidence of radiographic progression as assessed in soft tissue per Response Evaluation Criteria in Solid Tumors (RECIST) 1.1 or in bone per PCWG2/3, or death from any cause, whichever occurred first.

Baseline patient characteristics across the 11 RCTs were generally consistent with respect to age (range: 69 to 72 years), proportion with Gleason score ≥8 (range: 48% to 70%), and ECOG ≤ 1 (range: 99% to 100%). Variation in time since initial prostate cancer diagnosis was noted which was likely due to varying start and end times for this variable across trials (ie, from diagnosis to randomization versus from diagnosis to first dose). Where reported, median time since initial diagnosis of prostate cancer ranged from 2.52 to 5.64 years. Variation in median baseline prostate-specific antigen (PSA) levels was also noted, which ranged from 16.8 to 62.5 μg/L. Most trials reported at least 60% Caucasian participants, except for NCT02294461 which reported 0% Caucasian participants. This trial was conducted solely in Asia, with sites in mainland China, Korea, Taiwan, and Hong Kong. The proportion of patients with BPI-SF score ≤ 3 was generally consistent, with most trials reporting more than 90% of patients with BPI-SF ≤ 3. PROpel had the lowest proportion of patients with BPI-SF ≤ 3 (71.1% and 78.1% in each arm). The proportion of patients with bone and visceral disease were difficult to compare due to variable methods of reporting across the included trials (Table 2).

**Table 2.** Summary of baseline patient characteristics for the included trials.

Trial; NCT	Arm (*n* patients)	Median age (years)	Median time since diagnosis (years)	Caucasian/white (%)	ECOG PS 0-1 (%)	Gleason ≥ 8 (%)	BPI-SF ≤ 3 (%)	Bone metastases (%)	Visceral/soft tissue metastases (%)	Median baseline PSA (μg/L)
**ACIS; NCT02257736^26^**	Apalutamide plus abiraterone acetate (*n* = 492)	71	4.9^a^	74	100	NR	96	Bone: 83	Adrenal gland, lung and liver: 15	32.3
									Soft tissue: 12	
								Bone only: 42		
									Lymph node: 48	
	Abiraterone acetate (*n* = 490)	71	4.0^a^	76	100	NR	96	Bone: 87	Adrenal gland, lung and liver:	31.2
									14 Soft tissue: 14	
									Lymph node: 47	
								Bone only: 42		
**Alliance A031201; NCT01949337^33^**	Enzalutamide (*n* = 657)	NR	NR	83	100	48	NR	83	Liver: 5	23.8
									Lung: 12	
									Nodal: 46	
	Enzalutamide plus abiraterone acetate (*n* = 654)	NR	NR	83	100	54	NR	82	Liver: 4	24.3
									Lung: 11	
									Nodal: 50	
**COU-AA-302; NCT00887198^23^ ^,^ ^34^**	Abiraterone acetate (*n* = 546)	71	5.5^b^	NR	NR	54	98	Bone only: 51	Soft tissue or node: 49	42
	Placebo (*n* = 542)	70	5.1^b^	NR	NR	50	97	Bone only: 49	Soft tissue or node: 50	37.7
**PEACEIII; NCT02194842^32^**	Radium-223 plus enzalutamide (*n* = 222)	70	NR	NR	NR	NR	NR	NR	NR	25.3
	Enzalutamide (*n* = 224)	70	NR	NR	NR	NR	NR	NR	NR	23
**ERA 223; NCT02043678^27^**	Radium-223 plus abiraterone acetate (*n* = 401)	71	NR	71	99	61	94	NR	NR	30
	Abiraterone acetate (*n* = 405)	71	NR	70	99	58	92	NR	NR	31
**IPATential150; NCT03072238^28^**	Ipatasertib plus abiraterone acetate (*n* = 547)	69	2.90^c^	69	100	61	NR	84	Lung or liver: 12	21.3
									Lymph node: 37	
	Placebo plus abiraterone acetate (*n* = 554)	70	2.80^c^	70	100	65	NR	84	Lung or liver: 12	29.3
									Lymph node: 42	
**KEYNOTE-641; NCT03834493^31^,d**	Pembrolizumab plus enzalutamide (*n* = 245)	71	NR	NR	99	NR	NR	Bone: 85.7	Visceral: 12.2	NR
								Bone only: 50.2	Liver: 3.5	
	Enzalutamide (*n* = 242)	70	NR	NR	99.7	NR	NR	Bone: 87.5	Visceral: 12.7	NR
								Bone only: 48.8	Liver: 5.1	
**NCT02294461^24^**	Enzalutamide (*n* = 202)	71	2.52^c^	0	100	69.7	100	93.9	Lung or liver: 12.1	56.2
									Lymph node: 27.3	
									Other soft tissue: 24.2	
	Placebo (*n* = 193)	71	2.57^c^	0	100	61.6	100	92.6	Lung or liver: 8.4	62.5
									Lymph node: 24.2	
									Other soft tissue: 27.4	
**PREVAIL; NCT01212991^25^ ^,^ ^48^**	Enzalutamide (*n* = 872)	72	5.64^e^	76.7	100	50.6	98.2	Bone only: 39.9	Lung or liver: 11.2	54.1
									Lymph node: 50.1	
	Placebo (*n* = 845)	71	5.38^e^	77.5	100	52.4	98.7	Bone only: 39.6	Lung or liver: 12.5	44.2
									Lymph node: 51.4	
**PROpel; NCT03732820^29^**	Olaparib plus abiraterone acetate (*n* = 399)	69	2.80^a^	71	99.9	66.4	71.1	87.5	Distant lymph nodes: 33.3	17.9
									Locoregional lymph nodes: 20.6	
									Lung: 10.0	
									Liver: 3.8	
	Placebo plus abiraterone acetate (*n* = 397)	70	3.29^a^	69	99.7	65	78.1	85.4	Distant lymph nodes: 30.0	16.8
									Locoregional lymph nodes: 22.4	
									Lung: 10.6	
									Liver: 4.5	
**TALAPRO-2; NCT03395197^30^**	Talazoparib plus enzalutamide (*n* = 402)	71	NR	60	100	70	NR	87	Lymph node: 37	18.2
									Lung: 11	
									Liver: 3	
	Placebo plus enzalutamide (*n* = 403)	71	NR	63	100	70	NR	85	Lymph node: 41	16.2
									Lung : 15	
									Liver: 4	

Abbreviations: BPI-SF = Brief Pain Inventory—Short Form; ECOG = Eastern Cooperative Oncology Group; mCRPC = metastatic castration-resistant prostate cancer; mHSPC = metastatic hormone-sensitive prostate cancer; PSA = prostate-specific antigen.

a Time from initial diagnosis to randomization.

b Time from initial diagnosis to first dose.

c Time since diagnosis.

d The subgroup of patients who had not received prior abiraterone acetate was used in the analysis to align with the population of interest. However, baseline characteristics reflect the overall trial population, which includes some patients who had received prior abiraterone in either the mCRPC or mHSPC setting.

e From initial diagnosis or first treatment of prostate cancer to randomization.

### Primary analysis

The primary analysis included RCTs jointly reporting rPFS and OS identified in the evidence base that met the PH assumption. Of the 11 trials in the evidence base, one trial (PEACEIII) failed to meet the PH assumption for OS and was therefore excluded. Consequently, a total of 9481 patients from 10 RCTs were incorporated to assess the correlation between rPFS and OS in the primary analysis. A summary of rPFS and OS inputs used in the analysis is presented in Table 3.

**Table 3.** Summary of efficacy outcomes for included trials.

Trial; NCT	Intervention	Comparator	rPFS HR (95% CI)	OS HR (95% CI)
**ACIS; NCT02257736^26^**	Apalutamide 240 mg	Placebo	0.70 (0.60, 0.83)	0.95 (0.81, 1.11)
	Abiraterone acetate 1000 mg	Abiraterone acetate 1000 mg		
	Prednisone 10 mg	Prednisone 10 mg		
**Alliance A031201; NCT01949337^33^**	Enzalutamide 160 mg	Enzalutamide 160 mg	0.86 (0.76-0.97)	0.89 (0.78-1.01)
		Abiraterone acetate 1000 mg		
		Prednisone 5 mg		
**COU-AA-302; NCT00887198^23^ ^,^ ^34^**	Abiraterone acetate 1000 mg	Placebo	0.52 (0.45, 0.61)	0.81 (0.70, 0.93)
	Prednisone 5 mg	Prednisone 5 mg		
**PEACEIII; NCT02194842^32^**	Radium-223 55 kBq/kg	Enzalutamide 160 mg	0.69 (0.54-0.87)	0.69 (0.52-0.9)
	Enzalutamide 160 mg			
**ERA 223; NCT02043678^27^**	Radium-223 55 kBq/kg	Abiraterone acetate 1000 mg	1.152 (0.960, 1.383)	1.195 (0.950, 1.505)
	Abiraterone acetate 1000 mg	Prednisone or prednisolone 5 mg		
	Prednisone or prednisolone 5 mg			
**IPATential150; NCT03072238^28^ ^,^ ^47^**	Ipatasertib 400 mg	Placebo	0.84 (0.71, 0.99)	0.90 (0.76, 1.07)
	Abiraterone acetate 1000 mg	Abiraterone acetate 1000 mg		
	Prednisone 5 mg	Prednisone 5 mg		
**KEYNOTE-641; NCT03834493^31^**	Pembrolizumab 200 mg IV Q3W	Placebo IV Q3W	1.14 (0.89-1.44)	1.02 (0.80-1.31)
	Enzalutamide 160 mg	Enzalutamide 160 mg		
**NCT02294461^24^**	Enzalutamide 160 mg	Placebo	0.31 (0.20, 0.46)	0.33 (0.16, 0.67)
**PREVAIL; NCT01212991^25^ ^,^ ^48^**	Enzalutamide 160 mg	Placebo	0.32 (0.28, 0.37)	0.73 (0.63, 0.85)
**PROpel; NCT03732820^29^**	Olaparib 300 mg	Abiraterone acetate 1000 mg	0.68 (0.57, 0.81)	0.81 (0.67, 1.00)
	Abiraterone acetate 1000 mg	Prednisone or prednisolone 5 mg		
	Prednisone or prednisolone 5 mg			
**TALAPRO-2; NCT03395197^30^ ^,^ ^49^**	Talazoparib 0.5 mg	Placebo	0.667 (0.551-0.807)	0.796 (0.661, 0.958)
	Enzalutamide 160 mg	Enzalutamide 160 mg		

Abbreviations: mg = milligram; kBq = kilobecquerel; kg = kilogram.

**Figure 1.**
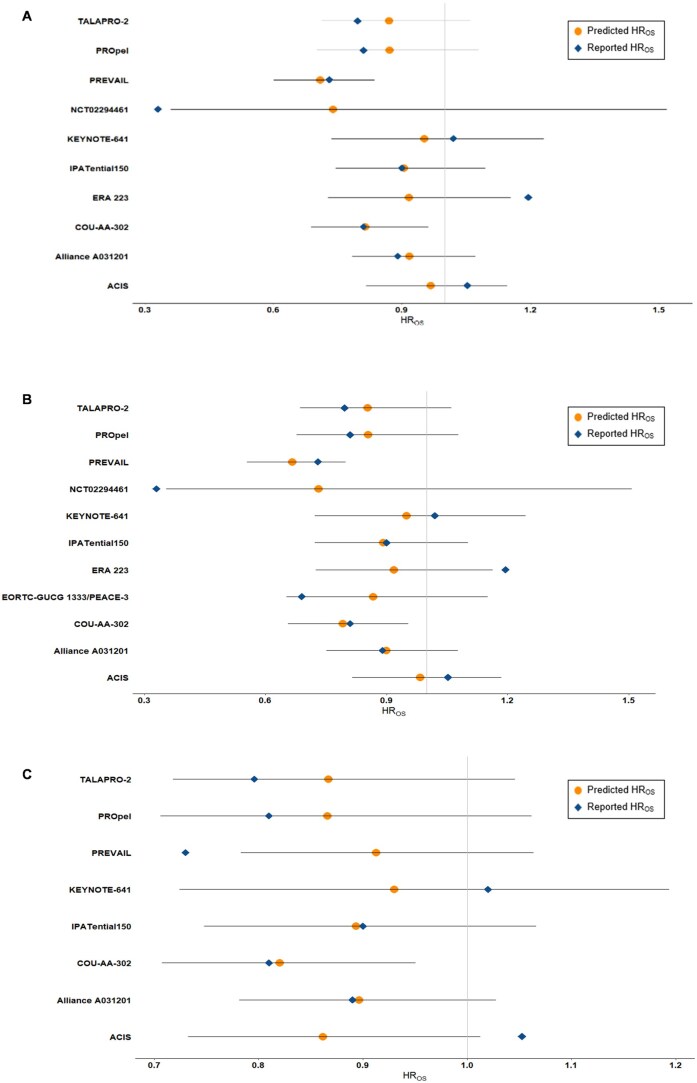
BRMA leave-one-out cross-validation. (A) Primary analysis excluding PEACEIII; (B) Sensitivity analysis including PEACEIII; and (C) Sensitivity analysis excluding PEACEIII, NCT02294461, and ERA 223. In the full analysis set, the BRMA for rPFS and OS from the 10 included trials reporting both outcomes had a R^2^ (95% CI) of 0.78 (0.53, 0.90) with a resulting correlation equation of log(HR_OS_) = −0.071 + 0.193 log(HR_rPFS_). In the first sensitivity analysis, the BRMA for rPFS and OS from the 11 included trials reporting both outcomes had a R^2^ (95% CI) of 0.69 (0.39, 0.87) with a resulting correlation equation of log(HR_OS_) = − 0.0769 + 0.218 log(HR_rPFS_). In the second sensitivity analysis, the BRMA for rPFS and OS from the eight included trials reporting both outcomes had a R^2^ (95% CI) of 0.92 (0.74, 0.97) with a resulting correlation equation of log(HR_OS_) = −0.0896 + 0.157 log(HR_rPFS_). Abbreviations: BRMA = bivariate random-effects meta-analysis; CI = confidence interval; HR = hazard ratio; OS = overall survival; rPFS = radiographic progression-free survival.

**Figure 2.**
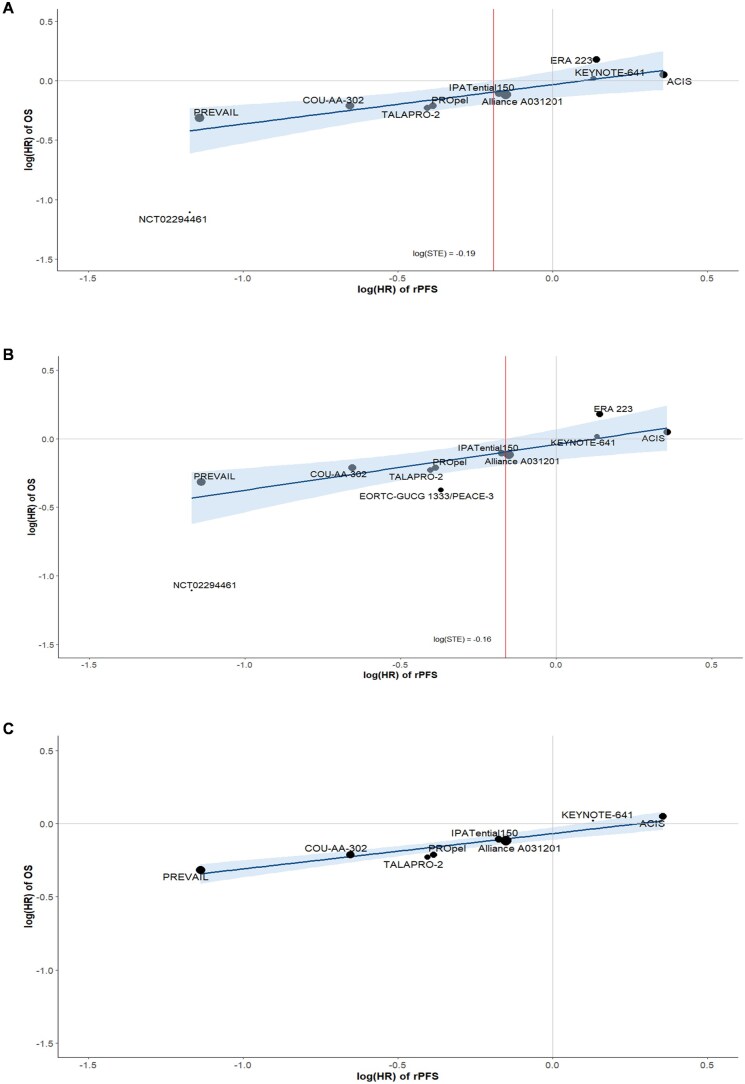
rPFS-OS Association: WLR. (A) Primary analysis excluding PEACEIII; (B) Sensitivity analysis including PEACEIII; and (C) Sensitivity analysis excluding PEACEIII, NCT02294461, and ERA 223. In the primary analysis, a scatterplot of reported log hazard ratio values for rPFS and OS from the 10 included trials had a R^2^ (95% CI) of 0.65 (0.40, 0.90), with a corresponding STE of 0.83. In the first sensitivity analysis, a scatterplot of reported log hazard ratio values for rPFS and OS from the 11 included trials reporting both outcomes had a R^2^ (95% CI) of 0.61 (0.35, 0.87), with a corresponding STE of 0.85. In the second sensitivity analysis, a scatterplot of reported log hazard ratio values for rPFS and OS from the eight included trials reporting both outcomes had a R^2^ (95% CI) of 0.91 (0.84, 0.99). Abbreviations: CI = confidence interval; HR = hazard ratio; OS = overall survival; rPFS = radiographic progression-free survival; STE = surrogate threshold effect; WLR = weighted linear regression.

According to IQWiG criteria, the trial-level analysis indicates a medium correlation between rPFS and OS (R^2^ = 0.78; 95% CI: 0.53, 0.90) based on the BRMA model, suggesting that 78% of the variability in OS effects can be explained by the observed effects on rPFS (Table 4). During LOOCV of the BRMA model, alignment between observed and predicted OS HRs was 80% in the primary analysis (Figure 1A). Using the WLR model, our analysis revealed a medium correlation between rPFS and OS, with an R^2^ value of 0.65 (95% CI: 0.40, 0.90) (Table 4 and Figure 2A). The resulting correlation equation from WLR was log(HR_OS_) = −0.032 + 0.332 × log(HR_rPFS_) and the corresponding STE was 0.83. It is therefore possible to draw conclusions on a significant effect in OS for a ­hypothetical trial demonstrating an upper confidence limit of HR_rPFS_ < 0.83 in rPFS.

**Table 4.** Summary of rPFS-OS correlation analyses.

Analysis	Number of studies	$R_{\mathrm{BRMA}}^{2}$ ** (95% CI)**	$R_{\mathrm{WLR}}^{2}$ ** (95% CI)**	Correlation strength (IQWiG criteria)	STE
**Primary analysis (excluding PEACEIII)**	10	0.78 (0.53, 0.90)	0.65 (0.40, 0.90)	Medium	0.83
**Sensitivity analysis 1 (including PEACEIII)**	11	0.69 (0.39, 0.87)	0.61 (0.35, 0.87)	Medium	0.85
**Sensitivity analysis 2 (excluding PEACEIII, NCT02294461 and ERA 223)**	8	0.92 (0.74, 0.97)	0.91 (0.84, 0.99)	High	NA

Abbreviations: BRMA = bivariate random-effects meta-analysis; CI = confidence interval; IQWiG = Institute for Quality and Efficiency in Health Care; NA = not applicable; OS = overall survival; rPFS = radiographic progression-free survival; STE = surrogate threshold effect; WLR = weighted linear regression.

Interpretation of correlation strength based on IQWiG criteria: A high correlation is indicated by a lower limit of the 95% confidence interval with R ≥ 0.85 (or R^2^ ≥ 0.72); a medium correlation is indicated by R values between 0.7 and 0.85 (or R^2^ values between 0.49 and 0.72); and a low correlation is indicated by an upper limit of the 95% CI with R ≤ 0.7 (or R^2^ ≤ 0.49).^14^

### Sensitivity analyses

Two sensitivity analyses were conducted to assess the robustness of the surrogate relationship between rPFS and OS. Sensitivity Analysis 1 included all 11 RCTs (*n* = 9927 patients), regardless of PH assumption violations. The BRMA model showed a medium correlation (R^2^ = 0.69, 95% CI: 0.39, 0.87), with 82% alignment between observed and predicted OS HRs using LOOCV (Table 4 and Figure 1B). The WLR model yielded a similar correlation (R^2^ = 0.61, 95% CI: 0.35, 0.87), and the resulting correlation equation of log(HR_OS_) = −0.041 + 0.334 × log(HR_rPFS_) and a STE of 0.85 (Table 4 and Figure 2B).

Sensitivity Analysis 2 excluded one trial violating the PH assumption (PEACEIII) and two visual outliers (NCT02294461 and ERA 223), resulting in 8 RCTs (*n* = 8287 patients). The BRMA model indicated a high correlation (R^2^ = 0.92, 95% CI: 0.74, 0.97), with 75% alignment between observed and predicted OS HRs using LOOCV (Table 4 and Figure 1C). The WLR model confirmed this (R^2^ = 0.91, 95% CI: 0.84, 0.99), with the resulting correlation equation: log(HR_OS) = −0.067 + 0.242 × log(HR_rPFS) (Table 4 and Figure 2C). Per IQWiG guidance, STE calculation was not required due to the high correlation.^14^

## Discussion

Overall survival remains the gold standard endpoint in RCTs for evaluating the efficacy of anti-cancer therapies. However, OS data often require long follow-up periods and may be confounded by post-protocol treatments, complicating interpretation. In contrast, rPFS offers a clinically meaningful, earlier endpoint unaffected by subsequent therapies. As an intermediate endpoint, rPFS can accelerate clinical development and regulatory decisions in mCRPC when a significant treatment effect is achieved and has been accepted by the FDA and the EMA as end point for registration of anticancer drugs.

This analysis evaluated the correlation between rPFS and OS using data from eleven RCTs evaluating first-line treatments for patients with mCRPC who had no prior exposure to ARPis in the first-line mCRPC setting. Results support rPFS as a valid surrogate for OS based on IQWiG criteria. The primary analysis demonstrated a medium positive correlation between rPFS and OS in the first-line mCRPC setting, consistent across two modeling approaches: BRMA (R^2^ = 0.78; 95% CI: 0.53, 0.90) and WLR (R^2^ = 0.65; 95% CI: 0.40, 0.90). Based on the corresponding STE, a trial with an upper confidence limit of HR < 0.83 for rPFS would be expected to show a significant OS benefit. Cross-validation reinforced the robustness of these findings.

Sensitivity analyses offered additional insight into the robustness of the rPFS-OS correlation. Inclusion of the PEACEIII trial, which violated the PH assumption, slightly reduced the strength of the correlation, though it remained within the medium range per IQWiG criteria. In contrast, excluding PEACEIII along with two outlier trials (NCT02294461 and ERA 223) resulted in a stronger correlation. Notably, these two outliers were the only studies that did not designate rPFS or OS as primary or co-primary endpoints, limiting their statistical power to detect treatment effects and likely contributing to their deviation from the overall trend. Their exclusion enabled a more consistent and reliable assessment of the rPFS-OS relationship.

Recent work by Vickers et al. compared six surrogacy models and highlighted important limitations in current surrogate endpoint studies, including the reliance on a single model and the lack of reporting of prediction intervals.^35^ The authors identified WLR and BRMA as useful models, with BRMA offering advantages by accounting for follow-up time and sampling error.^35^ Our analysis aligns with these recommendations by applying both WLR and BRMA models and reporting prediction intervals using LOOCV, strengthening the robustness and reliability of our findings.

To date, rPFS has not been formally established as a surrogate endpoint for OS in prostate cancer, and the current literature presents mixed evidence on the correlation strength between rPFS and OS in this setting. Chen et al. analyzed both RCT and real-world data in patients with castration-resistant prostate cancer (CRPC), finding rPFS to be a strong predictor of OS (adjusted R^2^ = 0.92) in trials of androgen-targeting agents such as abiraterone and enzalutamide, but not in taxane-treated populations.^36^ Halabi et al. evaluated post-docetaxel mCRPC patients and reported a moderate correlation (R^2^ = 0.65) using WLR, which fell short of their prespecified surrogacy threshold.^37^ Leung et al. focused on chemotherapy-naïve mCRPC patients and found similar moderate correlations (R = 0.66-0.67) using both WLR and BRMA.^38^ Woo et al. using RCT data and the PCWG2 definition of rPFS, reported a moderate correlation (R^2^ = 0.58) in a mixed mCRPC population.^8^ In contrast, Scher et al. analyzed progressive CRPC patients treated with taxane or epothilone therapies and found a weaker association (Kendall’s τ = 0.40), potentially due to interval censoring and early therapy discontinuation based on imaging changes that may not reflect true treatment failure.^39^

Differences in correlation strength across studies may be attributed to several factors, including variations in statistical methodology (eg, Spearman’s ρ and Kendall’s τ versus WLR), analytic approaches (eg, adjusted versus univariate analyses), patient populations (eg, treatment-naïve versus previously treated), drug classes (eg, ARPis versus taxanes), post-protocol treatments, and timing of assessments (eg, continuous OS measurement versus interval-based radiographic assessments). These factors may dilute the observed relationship between rPFS and OS.

Although there is currently no study which established rPFS as a valid surrogate endpoint in prostate cancer, the IQWiG criteria have been successfully applied to establish surrogate relationships in other malignancies.^40^^,^^41^ For example, in metastatic breast cancer, Lux et al. reported a medium trial-level correlation between progression-free survival (PFS) and OS (*r *= 0.72, 95% CI: 0.35, 0.90) using meta-regression, with a STE of 0.60.^41^ Similarly, Ajani et al. demonstrated a medium trial-level correlation using a BRMA model (R^2^ = 0.83; 95% CI: 0.70, 0.90; STE = 0.82) between disease-free survival and OS in resectable esophageal and gastroesophageal junction cancers.^40^

A key strength of this analysis was the comprehensive literature review, which captured recent advances in first-line mCRPC treatment and adhered to best practices for conducting and reporting systematic reviews to ensure transparency and reproducibility.^16^^,^^17^^,^^42^ To minimize heterogeneity, we systematically evaluated study and patient characteristics before conducting correlation analyses. Our strict inclusion of first-line, asymptomatic or mildly symptomatic mCRPC populations ensured greater certainty in our estimates. In contrast, other correlation studies included mixed populations or patients who were not truly treatment-naïve, or they were more symptomatic in terms of pain.^9^^,^^36^^,^^37^ The definitions of rPFS were consistent across the 11 included studies, all of which used RECIST 1.1 and PCWG2/3 criteria.^43-45^ Furthermore, by including the combination PARPi and ARPi therapies (eg, talazoparib plus enzalutamide and olaparib plus abiraterone acetate), this analysis reflects the most contemporary treatment landscape.

The results of this study should be interpreted within the context of the following limitations. First, not all studies in our evidence base assessed rPFS or OS as primary or co-primary endpoints. As a result, some trials (NCT02294461 and ERA 223) may have been underpowered to detect treatment effects for these outcomes. Including such studies may introduce variability in endpoint measurement and statistical power, potentially affecting the observed correlation between rPFS and OS. To address this, a sensitivity analysis was conducted to evaluate the impact of excluding these trials, and the overall findings remained robust. Second, this analysis was restricted to published summary-level data reported in RCTs, restricting our ability to assess correlations at the individual patient level. Access to individual patient data and the integration of real-world evidence could enhance understanding of the rPFS-OS relationship and improve generalizability to broader clinical settings. Third, while we explored the feasibility of conducting subgroup analyses by drug class (eg, PARPis, ARPis), the small number of studies within each class limited our ability to perform class-specific evaluations. Nevertheless, our LOOCV exercise predicted OS within the 95% prediction interval in nine of the eleven studies, suggesting that heterogeneity in drug class may not significantly impact the robustness of the observed association. Additionally, we acknowledge that the latest update of the SLR was conducted in August 2024. While we strive to include the most recent and relevant studies, the dynamic nature of clinical research means that literature reviews can quickly become outdated. We have made every effort to incorporate the latest data available at the time of writing. We also recognize that this is an evolving field that benefits from continuous updates. Notably, while the IPATential150 trial was included in our analysis, we are aware of updated OS data published in June 2025, which were not captured in our current evidence base.^46^ However, the updated HR for OS was very similar to the value used in our analysis (HR of 0.91 versus 0.90, respectively), suggesting the inclusion of the newer data would not meaningfully impact the results or alter the interpretation of the observed correlation between rPFS and OS.^46^^,^^47^ Finally, our correlation analyses are limited to patients with mCRPC receiving systemic treatments in the first-line ARPI naïve setting. Therefore, our results cannot be directly applied to different patient populations (ie, men with symptomatic disease, men treated with intensified therapy). Future research that includes broader patient populations will be necessary to determine whether the observed rPFS-OS relationship holds across different clinical settings.

While earlier access to life-prolonging therapies can offer meaningful benefits to patients, such approvals often occur before long-term clinical efficacy and safety data are available. To mitigate potential risks, several factors should be carefully considered, including the treatment indication and disease stage (eg, early- vs. late-stage), the nature of the therapy (eg, drug class, combination versus replacement), and the trial design (eg, superiority versus non-inferiority). For example, approvals based on early endpoints may warrant greater caution in early-stage disease, where patients have longer survival horizons, or in combination regimens that may carry additive ­toxicity. In contrast, replacement therapies, such as chemotherapy-sparing options that maintain efficacy while improving safety and quality of life, and treatments for late-stage disease, where prognosis is poor and curative options are limited, may be more readily accepted by clinicians and regulators.

## Conclusion

Our analyses suggested that rPFS is correlated to and a valid and useful surrogate predictor for OS in 1 L ARPI naïve asymptomatic or mildly symptomatic mCRPC for the treatments assessed. It is therefore possible to draw conclusions on a significant effect in OS for a hypothetical trial demonstrating an upper confidence limit of HR < .83 in rPFS according to IQWiG recommended methodology. Importantly, OS remains the gold standard clinical endpoint for determining the therapeutic benefit of treatments in oncology. While rPFS can serve as a valid and useful surrogate endpoint to approximate OS in 1 L mCRPC, it should not replace OS as the definitive measure of survival.

## Supplementary Material

oyaf425_Supplementary_Data

## Data Availability

The data underlying this article are available in the article and in its online supplementary material.
